# Steps Towards Developing Effective Treatments for Neuropsychiatric Disturbances in Alzheimer’s Disease: Insights From Preclinical Models, Clinical Data, and Future Directions

**DOI:** 10.3389/fnagi.2020.00056

**Published:** 2020-03-06

**Authors:** Amalie Clement, Ove Wiborg, Ayodeji A. Asuni

**Affiliations:** ^1^Laboratory of Neurobiology, Department of Health, Science and Technology, Aalborg University, Aalborg, Denmark; ^2^Department of Physiology and Symptoms, H. Lundbeck A/S, Copenhagen, Denmark

**Keywords:** Alzheimer’s disease, sleep disturbance, apathy, depression, neuropsychiatric disturbances, preclinical animal models

## Abstract

Alzheimer’s disease (AD) is the most common form of dementia worldwide. It is mostly known for its devastating effect on memory and learning but behavioral alterations commonly known as neuropsychiatric disturbances (NPDs) are also characteristics of the disease. These include apathy, depression-like behavior, and sleep disturbances, and they all contribute to an increased caregiver burden and earlier institutionalization. The interaction between NPDs and AD pathology is not well understood, but the consensus is that they contribute to disease progression and faster decline. Consequently, recognizing and treating NPDs might improve AD pathology and increase the quality of life for both patients and caregivers. In this review article, we examine previous and current literature on apathy, depressive symptoms, and sleep disturbances in AD patients and preclinical AD mechanistic models. We hypothesize that tau accumulation, beta-amyloid (Aβ) aggregation, neuroinflammation, mitochondrial damage, and loss of the locus coeruleus (LC)-norepinephrine (NE) system all collectively impact the development of NPDs and contribute synergistically to AD pathology. Targeting more than one of these processes might provide the most optimal strategy for treating NPDs and AD. The development of such clinical approaches would be preceded by preclinical studies, for which robust and reliable mechanistic models of NPD-like behavior are needed. Thus, developing effective preclinical research models represents an important step towards a better understanding of NPDs in AD.

## Introduction

Alzheimer’s disease (AD) remains a major cause of morbidity and mortality worldwide and is substantially burdensome to affected persons and their caregivers. It has been estimated that 47 million people worldwide were living with AD in 2015 (Alzheimer’s Disease International, 2015) and by 2060 15 million people will be diagnosed with AD in the US alone (Brookmeyer et al., 2018); thus, AD is a large social and financial burden to society.

The characteristic hallmarks of AD include β-amyloid (Aβ) plaques, neurofibrillary tangles (NFT), and neuroinflammation, and as the disease progresses almost all brain regions become affected with cell death and neuronal degeneration in the terminal stages eventually leading to substantial brain shrinkages and death. In addition to cognitive impairment, 80–97% of AD patients experience at least one non-mnemonic symptom at least once during the course of the disease (Gauthier et al., 2010; Van Dam et al., 2016; Tiel et al., 2019). These are denoted neuropsychiatric disturbances (NPDs) and include symptoms like apathy, depression-like behavior, sleep disturbances, aggression, and anxiety (Zhao et al., 2016). We have focused the discussion in this review on the three most common NPDs: apathy, depression-like behavior, and sleep disturbances. The factors that mediate the transition from prodromal AD to full-blown AD are not well characterized, but it is suggested that the onset, tempo, and rate of pathogenesis is impacted by significant contributions from NPDs (Geda et al., 2013; Zhao et al., 2016). Additionally, NPDs can appear before the prodromal phase of AD (Bartolini et al., 2004; Bidzan and Bidzan, 2014; Peters et al., 2015) and thus increase and prolong the caregiver burden; eventually, leading to earlier institutionalization (Steffens et al., 2005; Rea et al., 2014), which in turn increases the financial burden to society.

The diagnosing of AD is based on general health status, cognitive tests, cerebrospinal fluid (CSF) based biomarker assessment, magnetic resonance imaging (MRI), and positron emission tomography (PET) imaging. A CSF biomarker profile for AD includes low levels of Aβ42, reflecting Aβ plaque deposits, and high levels of total tau (T-tau) and hyperphosphorylated tau (P-tau), reflecting increased axonal damage and tau pathology, but other biomarkers have also been investigated to aid in early diagnosis, prognosis, and progression of AD (Blennow et al., 2010; Snyder et al., 2014; Tan et al., 2014). As NPDs have been shown to precede cognitive symptoms (Masters et al., 2015), they may represent additional biomarkers of early-stage AD and could enhance and accelerate the diagnosis of AD. However, few commonly accepted diagnostic tools for NPDs in AD exists. The Neuropsychiatric Inventory (NPI; Cummings et al., 1994; Cummings, 1997), and modifications of this (de Medeiros et al., 2010; Guercio et al., 2015; Morganti et al., 2018), is widely used to assess NPDs but it is not specific to AD and thus makes it difficult to use NPDs as biomarkers for early AD. The early and effective intervention of AD will improve quality of life for both patients and caregivers; therefore, recognizing and treating NPDs that may also contribute to AD pathophysiology (Geda et al., 2013; Zhao et al., 2016) is crucial. For this reason, we need to understand the underlying pathological mechanisms of NPDs and establish molecular biomarkers that will help in identifying and discriminating NPDs in early AD to support the development of better diagnostic tools and novel therapies.

## The Unmet Need for Addressing NPDs

The Alzheimer’s Association Research Roundtable gathered in 2010 and 2016 to address the issue of NPDs in AD and improve the understanding of the underlining mechanisms (Lyketsos et al., 2011; Lanctôt et al., 2017). NPDs decrease quality of life (Robert et al., 2010), increase caregiver burden (D’Onofrio et al., 2015), and are associated with faster decline and earlier institutionalization (Steffens et al., 2005; Rea et al., 2014). Thus, the conclusion of the gathering was that better treatments of NPDs in AD patients are of the highest importance and that adequate research is necessary to support such developments. In spite of this, clinical trials for treating NPDs report conflicting results, no superiority of drug over placebo, and/or accompanying higher risk of adverse events (Banerjee et al., 2011; Brandt and Pythtila, 2013; Wang et al., 2015; Leyhe et al., 2017). The use of antipsychotics in the elderly can additionally increase the risk of mortality; moreover, dementia accelerates this risk which has led to an FDA black-box warning (Maust et al., 2015; Schmedt et al., 2016). AD patients, in particular, experience psychotropic-related adverse events when compared to age-matched non-AD patients (Sepassi and Watanabe, 2019). This suggests that different molecular circuits are involved in the development of non-mnemonic symptoms in AD patients when compared to younger patients experience similar symptoms. It further underlines the urgency for better understandings of the etiology and pathology of NPDs in AD.

Model organisms have been important tools in the studies of AD (Hall and Roberson, 2012) but unlike memory and learning, which are reasonably well modeled in AD transgenic rodents (Webster et al., 2014), NPD-like behavioral changes are less characterized in such models. Thus, valid preclinical models for NPDs need to be developed to support drug discovery research and allow us to expand the knowledge of NPD pathology.

### Apathy and Depression-Like Behavior in AD

Apathy and depression can be difficult to distinguish and have overlapping symptomatology, like retarded executive function, and will be explored in parallel in this review. Apathy is the most common NPD in AD patients (Van Dam et al., 2016; Zhao et al., 2016; Lanctôt et al., 2017) with an overall estimated prevalence of 49%, while depressive behavior has an estimated prevalence of 42% (Zhao et al., 2016). Despite their similarities, symptoms like guilt, sadness or hopelessness are only associated with depressive behavior (Nobis and Husain, 2018).

Apathy is classified as a neurocognitive disturbance and defined as a reduced motivation for at least 4 weeks complemented by two of the following behaviors: reduced goal-directed behavior, reduced goal-directed cognitive activity, and emotional flattening (Van Dam et al., 2016). Meanwhile, apathy correlates with the severity of AD (Tschanz et al., 2011) and has been shown to persist if left untreated with a stronger association with mortality (van der Linde et al., 2017). On the other hand, depressive symptoms are more closely associated with reduced activity of daily living (ADL) and more serious aggression and wandering in AD patients compared to AD patients without depressive symptoms (Lyketsos et al., 1997). Finally, depressive symptoms accelerate cognitive decline in mild cognitive impairment (MCI; Brendel et al., 2015), while apathy predicts the transition from cognitively normal to MCI to AD (Guercio et al., 2015). In combination, patients with both apathy and depressive behavior are less independent and have lower ADL compared to AD patients with only apathy, depressive behavior, or none of the symptoms (Benoit et al., 2012). Thus, recognizing, discriminating, and treating apathy and depressive behavior are important but in AD pharmacological treatment of apathy (Rea et al., 2014) and depressive symptoms (Orgeta et al., 2017) have proven difficult; most likely, explained by the lack of knowledge about circuits involved in the symptom development.

#### Pharmacologic Interventions

Methylphenidate, which increases dopamine, norepinephrine (NE), and other catecholamines in the brain, ameliorates apathy in both mild (Padala et al., 2018) and moderate (Herrmann et al., 2008; Rosenberg et al., 2013) stages of AD compared to placebo. Although the studies differed on apathy rating scales and length of treatment, it suggests that dopamine and/or NE are relevant for the molecular circuits involved in apathy in AD. Supporting this, several studies have mapped apathy to specific brain regions with abnormalities including the anterior cingulate cortex, the prefrontal cortex, and the basal ganglia (Stella et al., 2014; Theleritis et al., 2014; Le Heron et al., 2017), all of which are innervated by dopaminergic pathways. ^11^C-PiB-PET imaging revealed correlations between Aβ deposits and apathy in the right anterior cingulate cortex and the bilateral frontal cortex in apathetic AD patients (Mori et al., 2014), suggesting a direct link between Aβ pathology and apathy which might be further linked to loss of homeostasis in dopamine and/or NE. Lastly, Padala et al. (2018) reported cognitive and emotional improvements after methylphenidate treatment, which highlights the need to treat NPDs in AD. Cumulatively, these findings indicate that apathy is closely linked to the worsening of AD parameters and that ameliorating apathy by targeting the dopaminergic and/or norepinephrinergic circuits might improve core AD pathology.

Depressive symptoms in AD associate with cortical thinning which has been specified to the temporal and parietal regions (Lebedeva et al., 2014) and lower gray matter volume of the left middle frontal cortex (Hu et al., 2015). Reduced cerebral blood flow in the dorsolateral prefrontal area (middle frontal gyrus) has also been documented in AD patients with depressive symptoms (Akiyama et al., 2008; Levy-Cooperman et al., 2008; Terada et al., 2014). Most of these regions are innervated by projections of serotonergic neurons from the raphe nuclei (Charnay and Léger, 2010), and considering the role of serotonin in mood (Yohn et al., 2017) these structural damages to brain areas sensitive to serotonin might explain depressive symptoms. However, using selective serotonin reuptake inhibitors (SSRIs) to treat depressive symptoms in AD has had limited success. A recent meta-analysis of randomized controlled trials (RCT) for the use of antidepressants in AD patients indicated that antidepressants have no effect over placebo (Orgeta et al., 2017). This indicates that dysregulation of serotonin alone cannot explain the development of depressive symptoms in AD.

The structural brain changes observed in AD patients with depressive symptoms might also be explained by mitochondrial dysfunction. Studies using a rodent model of depression have shown impairment of oxidative phosphorylation (OXPHOS) possibly caused by changes in mitochondrial membrane integrity (Rezin et al., 2008; Gong et al., 2011) which ultimately can lead to apoptosis (Wang, 2001) and neuronal cell death and thereby structural brain changes. Mitochondrial damage has been reported in both AD (Swerdlow, 2018) and depression (Bansal and Kuhad, 2016) and both patient groups show reduced glucose metabolism using fluorodeoxyglucose (FDG) PET (Hunt et al., 2007; Wei et al., 2016; Rice and Bisdas, 2017; Fu et al., 2018). Supporting this, a *Ginkgo biloba* extract (GBE) that has free radical scavenging properties, enhances mitochondrial membrane potential, and increase ATP production (Lejri et al., 2019) revealed a significant effect on apathy and other NPDs in AD patients (Scripnikov et al., 2007). On the contrary, a recent RCT found no effect on NPDs when patients were treated with resveratrol which acts on several proteins important for mitochondrial function (Zhu et al., 2018). This indicates that specific mitochondrial pathways may, at least partly, drive depressive and apathetic symptoms in AD but more studies are needed to unravel these specific pathways. The positive effect of GBE might be driven by the free radical scavenging properties because reactive oxygen species (ROS) produced during electron transport chain and OXPHOS increase during mitochondrial damage and can induce neuroinflammation *via* NF-κB signaling pathways which in turn increases AD pathology (Kaur et al., 2015).

Lebedeva et al. (2014) found a negative correlation between cortical thickness and levels of CSF T-tau and P-tau in AD patients with depressive symptoms which were not observed for CSF Aβ42 and suggests that only tau pathology is linked to depressive symptoms in AD. Although studies on tau pathology and depressive symptoms in AD are limited, a recent study reported that Braak stage I/II scores (NFT in entorhinal cortex and hippocampus) in post-mortem AD patients was significantly associated with depressive behavior along with other NPDs (Ehrenberg et al., 2018). On the other hand, MCI patients with depressive symptoms had higher amyloid pathology in frontotemporal and insular cortices compared to MCI patients without depressive symptoms which further correlated to a faster cognitive decline (Brendel et al., 2015). Altogether, these studies indicate that depressive symptoms in AD might be unrelated to the serotonergic system and that AD-related pathology causes damage to specific brain regions resulting in the development of depressive symptoms. Mapping how such pathological damage is mediated in relation to depressive symptoms represents an important task in the development of novel treatment options for depression in AD.

### Sleep Disturbances in AD

Thirty-nine percent of AD patients experience sleep disturbances (Zhao et al., 2016) and these cover a broad range of altered sleep-wake patterns including fragmented sleep, excessive daytime sleepiness, trouble falling asleep or maintaining sleep, and early morning awakening (Suzuki et al., 2017). Although it is unclear what drives sleep disturbances in AD, substantial evidence suggest that they significantly contribute to early pathological development (Spira et al., 2014; Kabeshita et al., 2017) and progression of disease (Mander et al., 2016; Musiek and Holtzman, 2016) and for this reason sleep disturbances have been investigated as a possible target for AD interventions (Mander et al., 2016).

Sleep disturbances can occur years before clinical AD symptoms (Spira et al., 2014; Kabeshita et al., 2017). Recently, a large systemic meta-analysis on sleep disturbances and risk of dementia showed that people with sleep disturbances at baseline have a 1.49 fold higher risk of developing AD compared to subjects without sleep disturbances (Shi et al., 2018). Alterations in sleep duration were also associated with an increased risk of cognitive decline (Chen et al., 2016) and ultimately dying from dementia (Benito-León et al., 2014). Similarly, Musiek et al. (2018) found that altered sleep patterns were associated with positive PiB-PET scanning in non-demented participants, underlining the link between sleep disturbances and AD pathology. However, age was also associated with circadian disruption and thus both age and AD pathology independently contributed to the association with sleep disturbances (Musiek et al., 2018). Supporting the link between age, sleep disturbances, and AD, Benedict et al. (2015) showed that 70-year old men with sleep disturbances have a 3-fold higher risk of developing AD compared to 70-year old men without sleep disturbances, while the risk of developing AD in 50-year old men was independent on sleep disturbances. Most AD patients are elderly and sleep disturbances are common in cognitively normal elder people too (Li et al., 2018); therefore, sleep disturbances in AD might be driven by age-dependent factors. The “mitochondrial cascade hypothesis” of sporadic AD postulate that mitochondrial dysfunction is the major cause of AD pathology and that Aβ accumulation is a secondary event (Swerdlow and Khan, 2004; Swerdlow et al., 2014; Swerdlow, 2018). Mitochondria are sensitive to aging due to lack of DNA repair mechanisms and thus mutations in mitochondrial DNA (mtDNA) accumulate over time (Grimm and Eckert, 2017). Consequently, mitochondrial dysfunction will increase with age and may represent one explanation for sleep disturbances in the elderly. Supportive of this view, Adler et al. (2020) found that aging disrupts the circadian rhythm in mice shown by loss of rhythmicity in proteins involved in circadian function. These proteins were linked to pathologies like AD and Parkinson’s Disease but also glycolysis and TCA cycle pathways (Adler et al., 2020) which are central to the electron transport chain and OXPHOS to produce ATP. Altogether, the reason for sleep disturbances to be so common in AD patients may be caused by age-dependent changes in mitochondrial function which might be even more severely compromised with AD pathology.

On the contrary, cognitively normal middle-aged people revealed that lower CSF Aβ42 was associated with worse objective sleep quality (Ju et al., 2013) and in 40-65-year old cognitively normal people self-reported worse sleep adequacy was associated with lower CSF Aβ42/Aβ40, higher CSF T-tau/Aβ42, and higher CSF P-tau/Aβ42 (Sprecher et al., 2017). Additionally, experimentally sleep-deprived healthy adults increased their Aβ production overnight with 25–30% compared to normal sleeping controls (Lucey et al., 2018) and the Aβ burden increased in the hippocampus and thalamus after one night of sleep deprivation in healthy individuals (Shokri-Kojori et al., 2018). These studies indicate that sleep disturbances can affect the levels of Aβ in the brain which might be due to increased production (Lucey et al., 2018) or decreased clearance of Aβ (Iliff et al., 2012), as described in the glymphatic system pathway (Plog and Nedergaard, 2018; Benveniste et al., 2019), or a combination of both. The default mode network (DMN) is active during awake non-task specific activities and inactive during sleep (Spreng et al., 2010). When activated it has a high neuronal activity which results in increased production of Aβ (Bero et al., 2011). Lastly, melatonin (the endogenous sleep-promoting hormone) acts on the DMN in an inhibitory manner (Zisapel, 2018) and AD patients have very low levels of melatonin which correlate with disease progression (Zhou et al., 2003). Thus, one explanation for the increased levels of Aβ with sleep deprivation could be increased activation of the DMN. Moreover, melatonin has several protective properties (for a more detailed description see Vincent, 2018) including the promotion of anti-inflammatory pathways and inhibition of pro-inflammatory pathways (Deng et al., 2006; Hardeland, 2018). The decrease of melatonin in AD patients could therefore both cause sleep disturbances and induce neuroinflammation both of which contribute to AD pathology.

The locus coeruleus (LC) is implicated in controlling wakefulness and arousal by the release of NE with high levels of neural activity during wakefulness and low activity during sleep (Aston-Jones and Bloom, 1981; Mitchell and Weinshenker, 2010). The LC neurons project to a variety of brain areas and networks and have anti-inflammatory effects (Feinstein et al., 2016; Giorgi et al., 2019) but the neurons and thus NE release is compromised in MCI (Grudzien et al., 2007) and AD brains (Zarow et al., 2003; Braak et al., 2011; Mravec et al., 2014). Studies have shown that microglia respond to Aβ42 fibrils in a pro-inflammatory manner which is abolished in the presence of NE (Heneka et al., 2010), thus increased levels of Aβ in sleep disturbances may contribute to neuroinflammation which cannot be suppressed in MCI or AD brains due to loss of the LC-NE system. Furthermore, these studies found that depletion of NE by the degradation of LC neurons caused increased Aβ deposits in the hippocampus and increased levels of Aβ42 but not Aβ40 in APP transgenic mice, suggesting that NE depletion causes changes in clearance rather than the production of Aβ peptides (Heneka et al., 2010). Altogether, these studies provide evidence of a partial explanation of how sleep disturbances accelerate AD pathology *via* the neuroinflammatory response to depletion of NE.

Interestingly, tau pathology has been reported in LC neurons in both children and young adults (Mather and Harley, 2016) but become significantly more pathological in MCI and early AD cases (Grudzien et al., 2007). It is further postulated that tau pathology spreads from the LC to other brain areas (Iba et al., 2015) and that oligomeric tau induces mitochondrial membrane leakage and subsequently loss of OXPHOS and mitochondrial biogenesis (Camilleri et al., 2020). Lastly, dysregulation of the LC-NE system associate with depressive symptoms, apathy, and sleep disturbances in AD (Matthews et al., 2002). A recent study showed clear synergistic toxicity of tau and Aβ with both increased neurodegeneration and behavioral changes in *C. elegance* (Benbow et al., 2020). It is, therefore, reasonable to think that sleep disturbances, and other NPDs, are not driven by a single pathologic event; however, further studies are warranted to substantiate this hypothesis.

#### The Molecular Mechanisms of Sleep

The circadian system is the foundation of the sleep-wake cycle and in both humans and rodents, the system is regulated by the suprachiasmatic nucleus (SCN) of the hypothalamus (Johnston et al., 2016), which receives light/dark inputs *via* the intrinsically photosensitive retinal ganglion cells containing melanopsin (Paul et al., 2009). However, nearly all cells in the body contain a circadian clock where the circadian oscillations are generated by a negative feedback loop. This loop consists of the core transcriptional activators CLOCK and BMAL1 (also known as ARNTL), who control the transcription of PER and CRY genes among others. The SCN neurons project to different areas of the hypothalamus in a complex manner and these projections are responsible for the circuit activity of neurotransmitters and neuropeptides that regulate the sleep/wake cycle. These include melatonin, serotonin, NE, acetylcholine, glutamate, GABA, dopamine, orexin, neurotensin, vasopressin, and vasoactive intestinal peptide (VIP; Lim and Szymusiak, 2015; Van Erum et al., 2018). A detailed description of the circuits is beyond the scope of this review.

The circadian system network is compromised in AD (Van Erum et al., 2018). The ventrolateral preoptic nucleus of the hypothalamus is innervated by SCN GABAergic neuron projections (Chou et al., 2003) and is important for maintaining sleep (Lu et al., 2000). AD patients have fewer neurons in this area (Lim et al., 2014), suggesting a link between AD pathogenesis and dysregulated sleep. The ventrolateral preoptic nucleus produces orexins (also known as hypocretins) and regulates wakefulness (Sakurai et al., 1998; de Lecea et al., 1998) and its neurons project predominantly to the LC and raphe nuclei (Peyron et al., 1998; España and Scammell, 2011). Compared to non-demented subjects, patients with moderate to severe AD have significantly higher CSF orexin levels associating with impaired nighttime sleep (Liguori et al., 2014). These levels correlated positively with CSF T-tau and CSF P-tau, while the Mini-Mental State Examination (MMSE) score correlated positively with sleep efficiency (Liguori et al., 2014). Orexin has also been linked to higher levels of soluble Aβ in an AD transgenic mouse model (Kang et al., 2009). Lastly, Liguori et al. (2017) found a significant increase in CSF orexin in AD patients and that hypothalamic glucose consumption correlated negatively with the CSF T-tau/Aβ42 ratio suggesting a causative link between mitochondrial function, AD pathology, and orexin. Although hypothalamic glucose consumption did not correlate with CSF orexin levels, the authors argue that the hypothalamus is compromised by AD pathology and this may cause the sleep disturbances in AD patients (Liguori et al., 2017). Altogether, this is supportive of orexin dysfunction as a viable target for AD intervention. In this regard, a phase III clinical trial (NCT02750306) to treat insomnia in patients with AD using the orexin receptor antagonist (Suvorexant) was effective and generally well-tolerated (Herring et al., 2019).

### Preclinical Research on NPD Related Behavior

Preclinical models are important tools for understanding pathological processes and developing novel treatment regimens; nevertheless, robust preclinical models for NPDs in AD are lacking. Although 189 AD transgenic rodent models exist today^1^, studies specifically characterizing NPD-like behavior in these models are limited.

Wister rats with oligomeric Aβ injected into the CA1 region of the hippocampus showed increased anxiety and memory impairment compared to vehicle injected rats without affecting hippocampal integrity (Salgado-Puga et al., 2015). This suggests, that Aβ oligomers trigger anxious behavior and memory impairment *via* cellular processes. In fact, others have shown that intracerebroventricular injected Aβ aggregates induce memory impairment and increased hippocampal levels of ROS and proinflammatory cytokines (Gupta et al., 2018). This indicates that Aβ oligomer injection to the brain can serve as a mechanistic model of NPD-like behavior. Double knockout PS1 and PS2 (DKO) mice experience age-dependent apathy shown by decreased nest building (Filali et al., 2009; Jirkof, 2014) activity compared to control littermates (Yan et al., 2013). Interestingly, this mouse model does not develop plaque or tangle pathology (Saura et al., 2004) but exhibit increased neuroinflammatory markers in the neocortex and hippocampus (Beglopoulos et al., 2004) and cortical neuron loss (Wines-Samuelson et al., 2010) at ages tested in the Yan-study. This suggests that neuroinflammation and/or neuronal loss can drive apathetic behavior unrelated to Aβ and tau toxicity. The 3xTg-AD mouse model shows anxiety and depressive behavior at ages where both Aβ and tau pathology is present. Interestingly, these behaviors could be ameliorated by melatonin treatment and protein changes on the protein levels were also normalized after treatment (Nie et al., 2017). Additionally, melatonin treatment significantly reduced the number of Aβ aggregates and neuroinflammation in 5xFAD mice (Jürgenson et al., 2019) and increased mitochondrial biogenesis together with a reduction in Aβ pathology in APPSwe/PS1dE9 mice (Song et al., 2018). Altogether, these studies indicate that the sleep promoting hormone, melatonin, is important for anxious and depressive behavior and that melatonin might act on both molecular and circuit pathways affected by Aβ pathology and neuroinflammation.

The APPSwe/PS1dE9 mouse model reported Aβ aggregation dependent disruption of the normal sleep-wake cycle with fewer sleep durations which could be rescued by preventing Aβ aggregation (Roh et al., 2012). These changes were accompanied by a significant reduction of Per1 and Per2 expression in the hippocampus and significant reduction of Per1, Per2, Cry1, and Cry2 expression in medulla-pons during the dark phase (Oyegbami et al., 2017). Similarly, APPxPS1 mice have decreased Per2 expression in the SCN at zeitgeber time (ZT) 2 and ZT10 (Duncan et al., 2012) while 5xFAD mice show significant changes in the mRNA expression patterns of both BMAL1 and Per2 in the SCN but these changes were less prominent on the protein level (Song et al., 2015). The McGill rats have increased expression levels of BMAL1 in the cortex and cerebellum but not in the hippocampus compared to age-matched control rats (Petrasek et al., 2018). Additionally, 3xTg-AD mice have reduced SCN neuron numbers and altered circadian rhythm (Sterniczuk et al., 2010) combined with lower numbers of norepinephrinergic neurons in the LC (Manaye et al., 2013).

SIRT1 is an NAD^+^-dependent deacetylase that is important for mitochondrial biogenesis (Wang and Chen, 2016) but also modulates the circadian cycle *via* its effect on the transcription of BMAL1 (Chang and Guarente, 2013). Serum levels of SIRT1 decrease with age and more dramatically with MCI and AD diagnosis (Kumar et al., 2013) and SIRT1 mRNA levels have been shown to decrease in an ApoE^−/−^ mouse model of AD together with loss of circadian rhythmicity compared to normal (C57BL/6J) mice (Zhou et al., 2016). These ApoE^−/−^ mice also had compromised mitochondria together with Aβ and tau pathology in the SCN (Zhou et al., 2016). Altogether, these studies provide evidence that dysfunctional mitochondria are implicated in the pathology of sleep disturbances in AD and that this implication might be triggered by obstruction of the normal function of SIRT1.

Altogether, these preclinical studies are consistent with the clinical data and provide evidence for a causal link between sleep disturbances, apathy, depressive behavior, and AD pathology. However, to substantiate these studies and our knowledge of NPD pathology we need to develop more robust preclinical models.

## Conclusion

**Figure 1:**
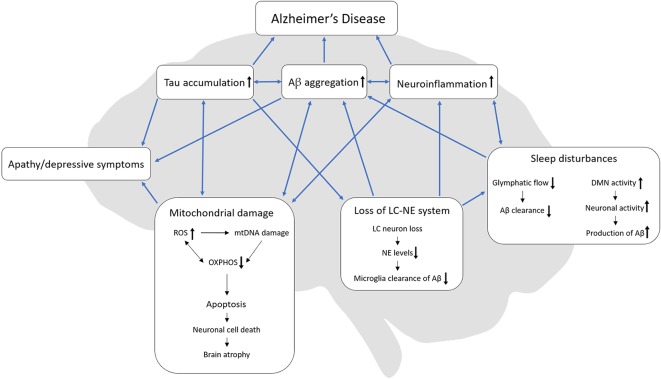
Interactions of AD-related pathologies, apathy, depressive symptoms, and sleep disturbances. We hypothesize, that loss of the LC-NE system might originate from tau accumulation in the LC which in turn can cause sleep disturbances. In turn, sleep disturbances can reduce the glymphatic flow and thereby decrease Aβ clearance. Also, sleep disturbances might increase the DMN activity due to the lack of sleep which then increases neuronal activity with a resulting increase in Aβ production. Both loss of the LC-NE system and sleep disturbances can increase neuroinflammation. During mitochondrial damage, ROS increases and can cause damage to both mtDNA and OXPHOS but ineffective OXPHOS can also increase the production of ROS. Mitochondrial damage can ultimately lead to apoptosis, neuronal cell death and lastly brain atrophy, which is present in late-stage AD. Both tau accumulation, Aβ aggregation and mitochondrial damage can lead to apathy/depressive behavior. AD, Alzheimer’s disease; Aβ, beta-amyloid; ROS, reactive oxygen species, OXPHOS, oxidative phosphorylation; mtDNA, mitochondrial DNA; LC, locus coeruleus; NE, norepinephrine, DMN, default mode network.

One notion put forward is that that NPDs appear because AD patients become unable to communicate or attend to their own needs and as a result feel misunderstood and become apathetic or depressive (Cohen-Mansfield et al., 2000). Although this hypothesis might explain some behavioral changes in AD patients, based on the literature reviewed here we propose a different hypothesis (summarized in Figure 1). Apathy, depressive behavior, and sleep disturbances are linked by pathophysiological events including mitochondrial damage, Aβ aggregations, tau accumulations, neuroinflammation, and loss of the LC-NE system, that together serve to drive and exacerbate AD progression. It is worth noting that separately, these pathophysiological processes are previous or current strategic targets for AD therapies, which have had limited success. Even though we still know little about these processes, targeting them in combination may prove to be the most optimal strategy and might pave the way for a better understanding of apathy, depressive symptoms, and sleep disturbances in AD; however, further investigations are needed to substantiate this hypothesis. Following this line of thinking, preclinical mechanistic models that allow us to dissect NPD processes are important. A strong commitment to building and studying such mechanistic models should be the next step towards developing and testing novel therapies in NPD research.

## Author Contributions

All authors contributed equally to the conceptualization of the manuscript. AC wrote the manuscript. AC, OW, and AA reviewed the manuscript.

## Conflict of Interest

AA is employed by the company H. Lundbeck A/S. AC is employed at both the company H. Lundbeck A/S and the University of Aalborg. OW declares that the research was conducted in the absence of any commercial or financial relationships that could be construed as a potential conflict of interest.

## Footnotes

^1^https://www.alzforum.org/research-models
